# Species Composition, Phenotypic and Genotypic Resistance Levels in Major Malaria Vectors in Teso North and Teso South Subcounties in Busia County, Western Kenya

**DOI:** 10.1155/2020/3560310

**Published:** 2020-01-25

**Authors:** Edward K. Githinji, Lucy W. Irungu, Paul N. Ndegwa, Maxwell G. Machani, Richard O. Amito, Brigid J. Kemei, Paul N. Murima, Geoffrey M. Ombui, Antony K. Wanjoya, Charles M. Mbogo, Evan M. Mathenge

**Affiliations:** ^1^Eastern and Southern Africa Centre for International Parasite Control (ESACIPAC) KEMRI, P.O. Box 54840-00200, Nairobi, Kenya; ^2^University of Nairobi, P.O. Box 30197-00200, Nairobi, Kenya; ^3^Machakos University, Machakos Campus, P.O. Box 136-90100, Machakos, Kenya; ^4^Centre for Global Health Research (CGHR) KEMRI, P.O. Box 1578-40100, Kisumu, Nyanza, Kenya; ^5^Vector-Borne Disease Control Unit, Ministry of Health, Nairobi Afya House, Cathedral Road, P.O. Box 30016-00100, Nairobi, Kenya; ^6^Jomo Kenyatta University of Agriculture and Training JKUAT Juja, P.O. Box 62 000-00200, Nairobi, Kenya; ^7^KEMRI-Wellcome Trust Research Programme, P.O. Box 43640-00100, 197 Lenana Place, Nairobi, Kenya

## Abstract

**Introduction:**

Knockdown resistance (*kdr*) is strongly linked to pyrethroid insecticide resistance in *Anopheles gambiae* in Africa, which may have vital significance to the current increased use of pyrethroid-treated bed net programmes. The study is aimed at determining species composition, levels of insecticide resistance, and knockdown patterns in *Anopheles gambiae* sensu lato in areas with and areas without insecticide resistance in Teso North and Teso South subcounties, Western Kenya.

**Materials and Methods:**

For WHO vulnerability tests, mosquito larvae were sampled using a dipper, reared into 3-5-day-old female mosquitoes (4944 at 100 mosquitoes per insecticide) which were exposed to 0.75% permethrin, 0.05% deltamethrin, and 0.1% bendiocarb using the WHO tube assay method. Species identification and *kdr* East gene PCRs were also performed on randomly selected mosquitoes from the collections; including adult mosquitoes (3448) sampled using standard collection methods.

**Results:**

*Anopheles gambiae* sensu stricto were the majority in terms of species composition at 78.9%. Bendiocarb caused 100% mortality while deltamethrin had higher insecticidal effects (77%) on female mosquitoes than permethrin (71%). Susceptible Kengatunyi cluster had higher proportion of *An. arabiensis* (20.9%) than resistant Rwatama (10.7%). Kengatunyi mosquitoes exposed to deltamethrin had the highest KDT_50_ R of 8.2. Both *Anopheles gambiae* sensu stricto and *Anopheles arabiensis* had equal S allelic frequency of 0.84. Indoor resting mosquitoes had 100% mortality rate after 24 h since exposure. Overall SS genotypic frequency in Teso North and Teso South subcounties was 79.4% against 13.7% homozygous LL genotype and 6.9% heterozygous LS genotype. There was a significant difference (*ρ* < 0.05) in S allele frequencies between Kengatunyi (0.61) and Rwatama (0.95). Mosquito samples collected in 2013 had the highest S allelic frequency of 0.87. *Discussion*. Most likely, the higher the selection pressure exerted indoors by insecticidal nets, the higher were the resistance alleles. Use of pyrethroid impregnated nets and agrochemicals may have caused female mosquitoes to select for pyrethroid resistance. Different modes of action and chemical properties in different types of pyrethroids aggravated by a variety of edaphic and climatic factors may have caused different levels of susceptibility in both indoor and outdoor vectors to pyrethroids and carbamate. Species composition and populations in each collection method may have been influenced by insecticide resistance capacity in different species. *Conclusions and Recommendations*. Both phenotypic and genotypic insecticide resistance levels have been confirmed in Teso North and Teso South subcounties in Western Kenya. Insecticide resistance management practices in Kenya should be fast tracked and harmonized with agricultural sector agrochemical-based activities and legislation, and possibly switch to carbamate use in order to ease selection pressure on pyrethroids which are useable in insecticidal nets and indoor residual spray due to their low human toxicity. The implication of such high resistance levels in mosquitoes collected in Teso subcounties is that resistance is likely to persist and or even increase if monomolecules of permethrin and deltamethrin or both continue to be used in all net- and nonnet-based mosquito control purposes. Usage of mutually reinforcing piperonyl butoxide (PBO) that prohibits particular enzymes vital in metabolic activities inside mosquito systems and has been integrated into pyrethroid-LLINs to create pyrethroid-PBO nets is an extremely viable option.

## 1. Introduction

African sub-Saharan zone accounted for 88% of all malaria cases in 2015 besides 90% of all deaths being attributed to malaria [1]. Fifteen countries in Africa contributed heavily to the global malaria burden in 2015 [2, 3]. Jointly, these nations recorded an estimate of 80 percent of worldwide malaria cases and 78 percent of deaths. Advances in reducing malaria incident globally in these countries with high burden have trailed behind other states. Malaria places a socioeconomic burden on African countries in addition to loss of human lives [3].

The four malaria parasite species of human *Plasmodium*, *P. vivax*, *P. ovale*, *P falciparum*, and *P. malariae*, are in Kenya [4]. Of the malaria positive slides, 96% were *P. falciparum* while 80 percent were unmixed infections and 16% were composite infections with *P. malariae* or *P. ovale* or both as recorded in KMIS 2010. Another 2% were unmixed *P. malariae* infectious agents and 1% was *P. ovale*. No *P. vivax* was detected in this survey [5]. The foremost malaria vectors in Kenya are adherents of *An. gambiae* complex and *An. funestus* complex [6].

Reduction in morbidity, aversion of mortality, and socioeconomic loss through incremental enhancement and reinforcing of national and local capacities for malaria intervention measures remain the fundamental aim for malaria control. In 2005, the President's Malaria Initiative (PMI) was started with an aim of decreasing deaths resulting from malaria by 50% in fifteen high-burden nations in African sub-Sahara [7]. A swift scale-up of four verified and greatly operational malaria deterrence and treatment approaches was envisaged: indoor residual spraying (IRS), long-term insecticidal mosquito nets (LLINs), precise *P. falciparum* detection and immediate management using artemisinin-based combination therapies (ACTs), and intermittent preventive treatment of pregnant women (IPTp) [8, 9]. The prime measures in the managing of malaria consist of timely detection and prompt management, indoor residual spraying (IRS), and the use of long-lasting insecticidal nets (LLINS). Regrettably, these strategic measures are pretty less operative due to the swift evolvement and proliferation of resistance against extensively utilized insecticides and drugs [10, 11]. Taking advantage of the genomic sequences of the malaria vectors and parasites may eventually result in new generations of insecticides and drugs, the upcoming of an efficient vaccine or genetically altered mosquitoes [12–14]. Unfortunately, these novel intervention measures may be inaccessible for the next ten years.

The necessity for efficacious measures in resistance regulation is becoming urgent as more insecticide-resistant species keep on rising globally, while insecticide resources are decreasing [15–17]. Projections for coming up with such approaches have improved due to recently upgraded information on ecology, biochemistry, molecular genetics, dynamics, monitoring, and other elements of resistance [18]. There are three fundamental approaches to insecticide resistance management: firstly, low selection pressure, supplemented by a strong component of nonchemical measures (that is, management by moderation); secondly, elimination of the selective advantage of nonvulnerable mosquitoes by increasing insecticide uptake through the use of attractants, or by suppression of detoxication enzymes through the use of synergists (that is, management by saturation); and thirdly, application of multidirectional selection by means of mixtures or rotations of unrelated insecticides or by use of chemicals with multisite action (that is, management by multiple attack) [19]. A season-long management program can be created from each aspect since these approaches are mutually inclusive. The measures considered in choosing must be the basis of one's extensive information on consequences on resistance. The approach selected should be based on knowledge of the resistance implications of the chosen insecticides and of the ecology and biology of the mosquito species while applying all possible nonchemical control measures [20, 21].

Controlling resistance is within reach due to the accessibility of more sensitive and user-friendly surveillance approaches [22, 23]. Use of insecticide-based strategies together with various noninsecticidal vector control approaches through integrated vector and pest management should be the first attempt towards resistance regulation [21, 24, 25]. Even though most of the programs intended for control can work properly during experimentation, they end up becoming impractical after scaling up to long-term intervention programs. Operationally, resistance management based on insecticide use is the most fundamental, and it can take a number of forms [26].

Malaria vector control option in Kenya has been faced with insecticide resistance in the main vectors, therefore a major concern for malaria vector control program managers [27]. Development of resistance is an evolving, complex, and dynamic process which is threatening to reverse gains in malaria control. Most commonly, when the frequency of resistant insects in a vector population increases, efficacy of the insecticide decreases up to the point where the insecticide has to be replaced by another one [19]. When vectors breed within or close to agricultural crops, they may be exposed to the same or similar insecticidal compounds, which will select for resistance [28]. Moreover, many insecticides are also extensively used to control domestic pests, further exerting more insecticide resistance selection pressure. Cross resistance causes vector populations to develop resistance very rapidly to newly introduced insecticides [29]. Moreover, insecticide resistance management (IRM) techniques such as rotations and mixtures can be undermined by issues of cross resistance [30]. The effectiveness of insecticide-based vector control is threatened as malaria mosquitoes develop resistance to the insecticides used in long-lasting insecticidal nets (LLINs) and indoor residual spray (IRS) [31]. Current efforts in global malaria control rely heavily on a single insecticide class: pyrethroids [32]. Insecticide resistance has therefore developed and has increased in distribution and intensity.

Mosquito and human habits, such as outdoor biting during late-night human activity, can also reduce the exposure of vectors to treated nets and sprayed walls [33]. Because LLINs and IRS play such a key role in malaria control programmes, these biological threats can potentially compromise the significant gains achieved through malaria vector control, and thus limit further success. Due to increase in resistance to pyrethroid insecticides, there is increasing concern that the benefits of LLINs could be lost altogether [30]. There is an urgent need to maintain the efficacy of a limited number of effective available insecticides. New tools to address mosquito resistance to insecticides are mostly in the early stages of development and evaluation [34]. Despite the huge investments in LLINs and IRS, many countries do not conduct routine malaria vector surveillance, including for insecticide resistance. Surveillance, monitoring, and evaluation and operational research are vital for tracking the progress of malaria prevention and control activities [35]. This study investigated species composition, resistance levels, and knockdown patterns among malaria vectors in Teso North and Teso South subcounties in Western Kenya.

## 2. Materials and Methods

### 2.1. Study Site

Teso North (represented by randomly selected Rwatama, Kengatunyi, and Akiriamasit clusters) and South (represented by randomly selected Kaliwa and Odioi clusters) subcounties are administrative areas in Busia County in western region of Kenya. Population at risk in Teso is 252,884 people. 16.9% of the population is aged between 0 and 4 years, 40.5% occupy 5 to 15 years' age bracket and 42.6% of the people are over 16 years. Teso subcounty and the Lake endemic region have 88.1% LLIN universal coverage. The main malaria vectors are *Anopheles gambiae* s.s., *Anopheles arabiensis*, and *An. funestus*. The region has malaria endemicity with year round transmission. The main malaria parasite is *Plasmodium falciparum*. It is a lowland located in the Lake Victoria Basin with an annual rainfall of around 1700 mm mean and minimum and maximum temperatures of about 17°C and 32°C, respectively [36, 37]. The communities mainly practice subsistence farming and health demographic characteristics include high infant mortality rates, neonatal and postnatal mortality rates, and crude death rates [38, 39].

Recent reports from Busolwe and Tororo Districts in Eastern Uganda near the border with Kenya indicate a high frequency of the *kdr* allele (1014S), similar to what has been observed in the Asembo study site, approximately 150-200 km to the southeast (Mawejje et al., 2013). However, the frequency of *An. gambiae* in mosquito collections is much higher than that observed in the Asembo study site and there are strong indications that the *kdr* allele is conferring both DDT and pyrethroid resistance particularly in homozygous-resistant individuals [40]. It was not clear whether this is the result of differences in ITN/LLINs ownership and use or whether resistance levels are higher in this area and leading to control failure. In addition, the impact of agricultural pesticides on insecticide resistance is unclear. Greater abundance of *An. gambiae* s.s., the high frequency of the *kdr* allele, and the evidence of phenotypic resistance in this region have led to the establishment of the study site on the Kenya-Uganda border in the subcounties of Teso. Given the close proximity of the current study site and districts in Uganda where resistance has been observed, it was interesting to investigate the *kdr* genotype and allele frequencies in Teso land. The study focused on characterization of species composition, resistance levels, and knockdown patterns in randomly selected clusters in Teso North and Teso South.

### 2.2. Study Design

The study design was a clustered complex longitudinal exploratory design where data was collected at several distinct periods on the same set of cases and variables. The purpose of the study was to describe patterns of change in species composition, resistance levels, and knockdown patterns. During baseline and two subsequent surveys, larvae were collected from the field and reared to produce *F*_0_ generation which was exposed to WHO tube susceptibility tests. *Kdr* gene and species identification was investigated among the exposed *F*_0_ generation and the adults collected using standard methods.

### 2.3. Sample Size Determination

Sample sizes were dependent on WHO assay requirements, whereby susceptibility tests were performed on nonblood-fed females, aged no more than 3-5-day post emergence. One hundred and fifty adult females were used, 100 of which were exposed to the insecticide (in 4 replicates each of around 25 mosquitoes). The remaining 50 served as “controls” (i.e., 2 replicates, each of around 25 mosquitoes). For positive control and negative control respectfully, 50 susceptible KISUMU Asembo strains of *Anopheles* mosquitoes were exposed in WHO tubes with insecticidal papers and 50 females exposed in WHO tubes with untreated papers. A sampling frame of household list from county registration files gave us the total number of households in each randomly selected sublocation or cluster, village, and compound, respectively. A clustered probability sample was achieved with the help of computer generated tables of random numbers which were used to select sublocation, village, compounds, and households where mosquito sampling was carried out. Selected houses lay within 2 km radius from larval collection sites. The sample size obtained was 96 houses, hence twenty houses per cluster or sublocation. Larval collection sites were randomly selected within 2-3 kilometres radius from selected households.

### 2.4. Mosquito Collection Methods

Both larvae and adult stages of mosquito were collected for two years after year one baseline susceptibility survey. Larvae were collected from their natural breeding sites using standard dippers, put in plastic containers, and transported to the laboratory for rearing, species identification, and susceptibility tests. Only *Anopheles* larvae were retained in the containers as screening was done on all collected larvae using morphological features described by [41]. Adult mosquitoes were collected using indoor resting vacuum aspiration, human landing catch, widow exit traps, pyrethrum spray catch method, and outdoor pot collection. Anopheline mosquitoes were identified morphologically as *An. gambiae sensu lato (*s.l.), *An. funestus*, other *Anopheles*, and non-*Anopheles* [41], and characterized by gonotrophic stata (empty, blood-fed, gravid, and half gravid female mosquitoes). The head and thorax were preserved in drierite and stored for use in sporozoite ELISA. Blood-fed abdomens were preserved in a freezer maintained at -18°C and stored for use in blood meal PCR. All legs and wings were preserved in drierite and stored for use in *kdr* gene and species identification. Adult sampling was done at the end of the long (May-July) and short (Oct-Nov) rains from 2012 to 2014.

### 2.5. Indoor Resting Vacuum Aspiration

A vacuum aspirator was used, either a motor driven one or a manually operated using suction pressure in the mouth. Collected adult mosquitoes were selected and put in paper cups and transported to the laboratory in a cooler box. Sucrose solution in moderately soaked cotton wool was placed on the paper cup net as food to the adult mosquitoes.

### 2.6. Window Exit Trap (WET)

To collect mosquitoes that bite indoors but rest outdoors in order to determine the effect of resistance on the normal movement and feeding habits of mosquitoes, window exit traps were used. In each study cluster, a total of five houses were randomly selected for window traps; an index house was randomly selected and additional houses were selected based on proximity to the index house. The window traps were placed over the windows of bedrooms at 6:00 pm. In the morning, the trapped mosquitoes were collected from the traps using a mouth aspirator. Window exit traps were most suitable for fitting only to rooms that are well sealed and that had few exit points for mosquitoes. Other openings other than the window to which the trap was fitted were covered or blocked with dark clothes or cartons except the eves. Usually, the sleeping room was selected and the trap well fitted to a window. Parts of the window not covered by the trap were covered with dark cloth, cartons, or hardboard. The trap was fitted in such a manner that the collecting sleeve pointed outward. It was important to fix traps into position well before sunset. Mosquito collection was done the following morning just after sunrise. All *Anopheles* mosquitoes were collected through the sleeve of the trap with the use of a mouth aspirator. Separate paper cups were used to transport the live and dead mosquitoes collected from each house. Household data was also entered in a structured questionnaire. The paper cups were clearly labelled in pencil or pen with at least the following essential information: location, date, exit trap number, house number or householder code, time of collection, whether mosquitoes were found dead or alive in the trap, and name of the collector.

### 2.7. Pyrethrum Spray Catch (PSC)

To obtain the indoor resting densities of mosquitoes, a pyrethrum spray collection method was done in all the houses that had window traps the previous night. Collection took place between 6 am and 8 am. Inhabitants in the house were asked to wait outside, during the procedure. The number of children and adults who reportedly slept in the house the previous night was recorded and the presence of ITNs/LLINs recorded. All food items and drinking vessels were removed from the house. White sheets were spread on the floor and over the furniture within the house. Two collectors, one inside the house and one outside, sprayed around the eaves with 0.025% pyrethrum emulsifiable concentrate with 0.1% piperonyl butoxide in kerosene. The collector inside the house then sprayed the roof and walls. The collector outside the house sprayed through the eaves ahead of the collector spraying inside the house. The house was closed for 10 minutes after which dead mosquitoes were collected from the sheets and transferred to the laboratory on moist filter paper inside petri dishes.

### 2.8. Human Landing Catch (HLC)

Female mosquitoes were attracted to humans as they quested for blood meals. The number of mosquitoes biting or landing on humans is a major determinant of malaria transmission. The suitable locations for the night collections were selected in such a way that they were closer to the vector breeding sites in the area. Direct collection of biting mosquitoes was performed during the night when malaria vectors were active for they take blood meal in the night. In a full-night programme, hourly collections were made during the entire period from 17 : 00 h to 07 : 00 h, therefore from dusk to dawn. Being a very laborious activity, two teams of collectors were used; each team working half of the night; both indoor and outdoor. In the case of outdoor collections, rainy hours when it was not possible to collect were also recorded. HLC was done for two consecutive nights per month, and the method involved a consented adult sitting down with legs exposed and waiting for mosquitoes to come feed on the collector where he or she used a mouth aspirator to collect the blood questing females and placed them in netted paper cups with a central upper hole blocked with cotton wool. Alupe Sub-District Hospital in the Teso South and Moding Health Centre in the Teso North subcounties provided medical supervision for all the collectors as for the other members of the community.

### 2.9. Outdoor Pot Collection (OPC)

Clay pots are often used for storing drinking water in the homes in the study area. The clay pots were locally designed, made, and placed outdoor from 18 : 00 to 06 : 00 h at about 5 m from the house [42, 43]. Each pot was about 20 litres capacity, with an opening of 20 cm width, a round bottom, and a maximum width of 45 cm. A 2 cm diameter hole was placed into the center of the base during manufacture. The hole made the pot useless to hold water, hence limiting likelihood of theft. Mosquitoes were collected from the pots once in the morning from 06 : 00 to 09 : 00 h by placing a cloth mesh from a standard adult mosquito cage on the opening and secured as described by [43]. One of the two samplers then lifted the pot to expose the opening to light and agitate mosquitoes inside, and blew into the small hole at the bottom, causing the mosquitoes inside the pot to take flight and enter the cage being held into position by the second sampler. The cloth mesh was then removed, and remaining mosquitoes in the pot were recovered with an aspirator and transferred to the cage, completing the collection.

### 2.10. Questionnaire Administration

Household information was collected from household heads in each of the houses where indoor resting catch, pyrethrum spray catch, and human landing catch were done. Data on the number of individuals who slept in the previous night, type of eaves, type of wall, type and ownership of net, frequency of net treatment, and indoor residual spraying was collected for each of the houses randomly selected.

### 2.11. DNA Extraction and Species Identification

DNA was extracted from the legs and wings of *An. gambiae* s.l. and *An. funestus* complexes using an ethanol precipitation method [44]. Conventional polymerase chain reaction (PCR) was used to distinguish between the two sibling species of the *An. gambiae* s.l. species complex native to Western Kenya, *An. gambiae* s.s. and *An. arabiensis* [45].

### 2.12. WHO Susceptibility Assays

Mosquito larva samples were reared and exposed to WHO susceptibility kits impregnated with 0.75% permethrin, 0.05% deltamethrin, and 0.1% bendiocarb insecticides. The WHO protocol was used for testing susceptibility to permethrin, deltamethrin, and bendiocarb insecticides. Treated test papers with the WHO diagnostic dosages were supplied by the WHO Collaborating Centre in Kenya. Cohorts of 25 female mosquitoes were exposed to different insecticides at temperatures of 25 ± 2°C and 70–80% relative humidity following the standard World Health Organization (WHO) tube test protocol [7]. Negative and positive controls were exposed to untreated and treated filter papers for 1 h, respectively. Knockdown time was recorded after every 10 minutes during the 60-minute exposure. After 1 h exposure, mosquitoes were transferred to recovery tubes and maintained on 6% sucrose solution for 24 h. Mortality was recorded after 24 h recovery period. Mosquitoes that were knocked down after 1 h exposure and those that were alive after the 1 h exposure and still surviving 24 h later were collected and stored individually in 95% alcohol for subsequent molecular analysis.

### 2.13. Genotyping for *Kdr* Mutations

DNA was extracted from adult *An. gambiae* and *An. arabiensis* mosquitoes as earlier described [45]. Real-time PCR was used to quantify the genotype at amino acid position 1014 of the voltage-gated sodium channel, following the methods of Bass et al., 2007, as modified by Mathias et al., 2011. Samples were genotyped for the wild-type (susceptible) allele using probe 5′-CTTACGACTAAATTTC-3′ and for the 1014S *kdr* allele using probe 5′-ACGACTGAATTTC-3′. Real-time (RT)-PCR reactions were done using Strategene MxPro 3000 machine using a 96-well format.

### 2.14. Data Collection, Management, and Analysis

WHO criteria for susceptibility are as follows: mortality rates between 98% and 100% indicate full susceptibility; mortality rates between 90% and 97% require further investigation while mortality rates < 90%, the population is considered resistant to the tested insecticides. Means in the experimental clusters were compared with the means in positive and negative controls as well as between clusters. Percent mortality values were corrected for control mortality using Abbott's formula [46]. The KD_50_ and KD_95_ were determined for each cluster and insecticide using probit analysis. All analysis was performed in SAS version 9.4 (SAS Institute, Cary, NC) [47]. The frequency of the resistance genotype and allele was calculated using the Hardy-Weinberg equilibrium test for *kdr* genotypes and *kdr* alleles.

## 3. Results

### 3.1. Species Composition

*Anopheles gambiae* sensu stricto were the majority in terms of species composition in both Teso North and Teso South subcounties and also in all the clusters randomly selected (Table 1). Highest proportions of *Anopheles gambiae* sensu stricto and *An. arabiensis* were found in Rwatama and Akiriamasit clusters, respectively. Only Teso North subcounty had traces of *Anopheles funestus*. Overall, the predominant *Anopheles gambiae* comprised of 78.9% of the female mosquitoes sampled in Teso subcounties. Highest proportion of *Anopheles gambiae* s.s. were collected using pyrethrum spray catch method while most *Anopheles arabiensis* were collected using a window exit method. Only outdoor pot collection method was able to sample *Anopheles funestus*.

### 3.2. Phenotypic Resistance Levels

Bendiocarb caused 100% mortality in 24 hours after exposure to the insecticide (Table 2). Deltamethrin was more potent than permethrin. Kengatunyi cluster mosquitoes were equally knocked down by both permethrin and deltamethrin. Mortality rates caused by permethrin as compared to deltamethrin were higher in 2012 but reversed in 2013 and 2014. All vectors were resistant to permethrin and deltamethrin but susceptible to bendiocarb.

### 3.3. Genotypic Resistance Levels

Rwatama cluster had significantly (*p* ≤ 0.05) higher proportion of SS genotypic and S allelic frequencies as compared to Kengatunyi cluster (Table 3). Teso South which is a bit farther from Kenya-Ugandan border had a higher S allelic frequency than Teso North which borders Uganda. All female mosquito samples collected through the pyrethrum spay catch method and tested for *Kdr* gene had a 100% SS genotypic frequency. There was no significant difference in allelic frequencies between *Anopheles gambiae* s.s. and *Anopheles arabiensis*. Mosquito samples collected in the year 2013 had the highest S allelic frequency.

Highest SS, LS, and LL genotypic frequencies were found in *Anopheles gambiae* sensu stricto collected in 2014 from the resistant Rwatama cluster, 2012 from susceptible Kengatunyi, and 2012 from susceptible Kengatunyi, respectively (Table 4). All *Anopheles arabiensis* collected from the resistant Rwatama had 100% SS genotypic frequency. Both *An. gambiae* sensu stricto and *An. arabiensis* collected from Teso South subcounty in 2013 had the same SS genotypic frequency.

### 3.4. Knockdown Patterns

**Figure 1:**
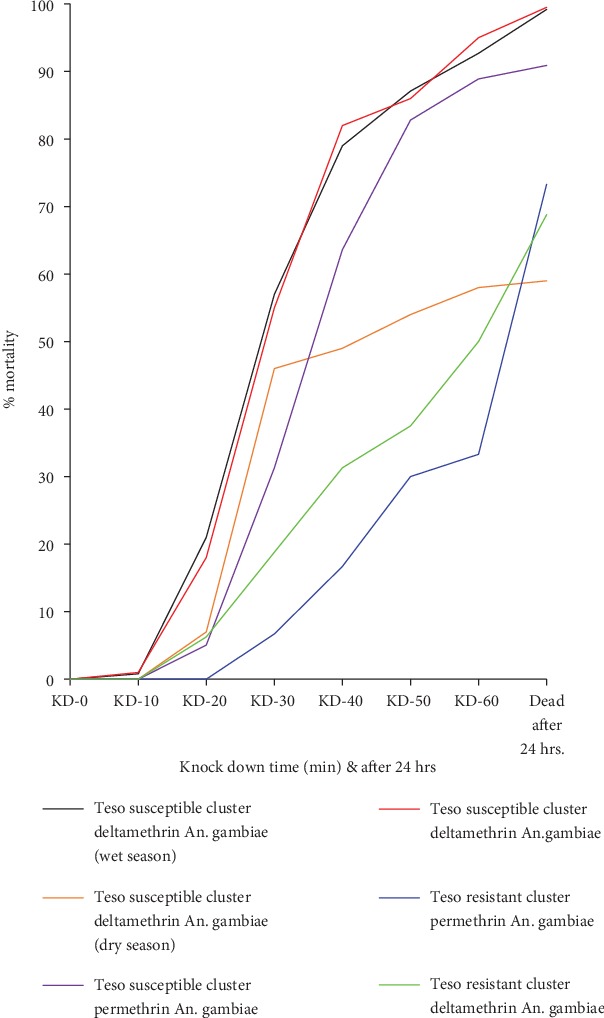
Knockdown curves showing the effects of different types of insecticides, wet or dry season, and resistance levels on knockdown trends in 3-5-day-old female mosquitoes collected in Teso North and Teso South subcounties, Western Kenya, between 2012 and 2014.

The knockdown patterns were dependent on resistance levels in the mosquitoes and the cluster, rainy or dry season, and the type of insecticide (Figure 1). Deltamethrin knocked down the female mosquitoes earlier and faster than permethrin. During dry season, the mosquitoes were not as readily knocked down as compared to mosquito samples collected during wet season.

The Kisumu Asembo *Anopheles gambiae* strain was fully susceptible to the three insecticides (Table 5). The knockdown times for 50% (KDT_50_) of mosquitoes sampled from Teso North and Teso South subcounties were between 24 and 47 min in contact with permethrin, 22 and 36 min with deltamethrin, and 20 and 25 min with bendiocarb. For the three insecticides, KDT_95_ were less than 190 min while less than 86% of exposed mosquitoes were dead at 24 h post exposure to permethrin and deltamethrin. Mosquitoes exposed to bendiocarb experienced 100% mortalities after 24 h post exposure. Susceptible Kengatunyi mosquitoes exposed to deltamethrin had the highest KDT_50_ R of 8.2.

### 3.5. Graphical Contrast between Phenotypic and Genotypic Resistance Levels

**Figure 2:**
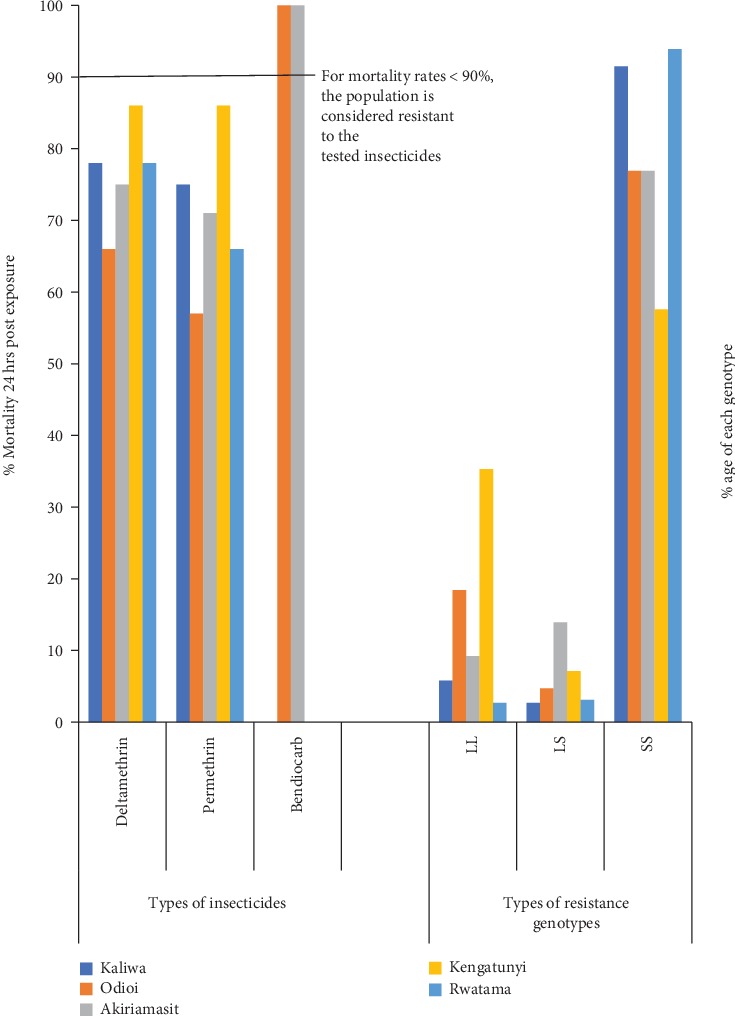
Phenotypic and genotypic resistance levels in *F*_0_*Anopheles gambiae* collected from five clusters in Teso North and Teso South subcounties, Western Kenya between 2012 and 2014.

Rwatama scored the highest levels of homozygous SS genotype for resistance at 93.9% while Kengatuny had the lowest levels of SS genotype at 57.6% (Figure 2). Vice versa, Kengatuny showed the highest levels of homozygous LL alleles for susceptibility at 35.3%. All clusters registered over 50% homozygous SS alleles. Akiriamasit had the highest levels of genetically transitioning heterozygous LS alleles at 13.9%. There was significant genotypic difference between resistance alleles in Rwatama and Kengatunyi (*ρ* < 0.05).

**Figure 3:**
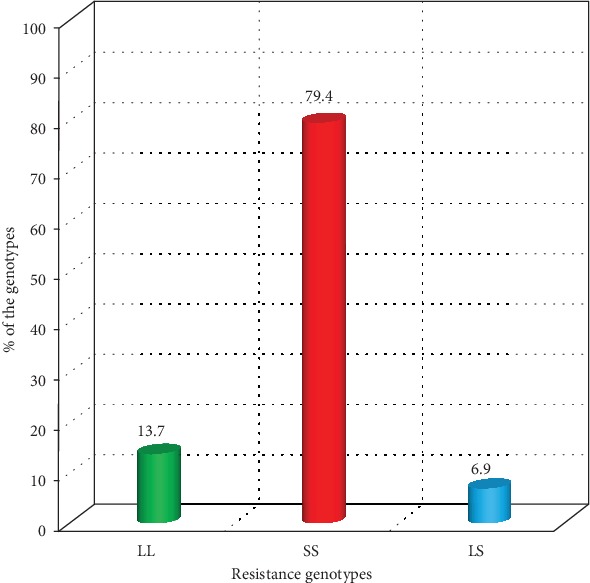
Teso subcounties' overall wild and mutant genotype levels in *F*_0_*Anopheles gambiae* collections sampled between 2012 and 2014.

Overall levels of homozygous-resistant, heterozygous-resistant, and homozygous-susceptible genotypic frequencies were 79.4, 6.9, and 13.7%, respectively (Figure 3). Transitioning LS genotype had the least proportion.

## 4. Discussion

*Anopheles gambiae* being the main malaria vector in Teso North as well as Teso South subcounties is very unique as several studies indicate that *Anopheles arabiensis* is the major malaria mosquito inside the Lake Victoria basin [48]. These malaria vectors breed in various environments stretching from short-term rain ponds to water bodies that are permanent. *Anopheles gambiae* breeds in temporary fresh shallow recently ploughed water pools and hoof prints with clean water. The study showed that *Anopheles funestus* and *Anopheles arabiensis* turned out as the minor *P. falciparum* vectors in Teso subcounties.

Apart from susceptibility to insecticidal agents in the environment, a number of factors such as anthropogenic undertakings, for example, development projects also govern distribution of vector species in East Africa. Further, climate-related atmospheric conditions, predominantly rainfall as well as temperature, have been considered as the dependent of habitations, hence vector distribution and abundance between high and low elevation regions. Distribution of vectors and parasites has well been facilitated by the movement and migration of people from high land to low land [49]. Furthermore, density and distribution of vector in all regions have largely been influenced by topography. Therefore, the density and distribution of proficient vectors have caused the ardent use of intensive interventions and control tools throughout the subregion, hence subsequent selection pressure.

A higher bulk of resistance alleles came from *An. gambiae* as opposed to *An. arabiensis* and *An. funestus* since the susceptible Kengatunyi cluster had higher proportions of *An. arabiensis* than the resistant Rwatama cluster. The rate of genes selecting for nonvulnerability to insecticidal chemicals depends on the species incriminated in a given area. *An. gambiae* was resting and feeding indoors, hence greater exposure to long-lasting insecticidal nets having pyrethroids on them. Most *An. arabiensis* and *An. funestus* were resting outdoor, thus minimal exposure to insecticidal nets. But still exophillic *An. arabiensis* and *An. funestus* had traces of resistance genes possibly due to the residual effect of insecticidal air emanating from air flow obstructing pyrethroid-treated nets or from agropesticides and domestic chemicals which contain insecticides as active ingredients. Because of its pressure of the vapor (1.5 × 10^−8^ mmHg at 25°C), deltamethrin has a low capability to volatilize hence possibly lower selection pressure as compared to permethrin (2.5 × 10^−8^ mmHg at 25°C). This may tend to explain why female mosquitoes were more vulnerable to deltamethrin than permethrin after 24-hour post exposure. [17], reported forced exophily that is premature escape of mosquitoes from the hut, deterrence, and knockdown by pyrethroid-treated bed nets. Depending on the type of insecticide used on nets and mosquito species, effects range from reduced house entry, reduced blood feeding success to greater likelihood of house exit [50, 51].

Deltamethrin was more lethal and quicker in knocking down female mosquitoes than permethrin. Both deltamethrin and permethrin may be used in long-lasting insecticidal nets while only deltamethrin can be exploited inside the house residual jetting for it has a longer indoor half-life. Pyrethroids are man-made biotic composites made from *Chrysanthemum* flowers that are widely utilized as commercial and household insecticidal agents. Since the pyrethroic and ketoalcoholic esters of chrysanthemic acid are lipophilic, they account for the insecticidal components through easy penetration into the insect body and immediate induction of toxicosis. Pyrethrins contain active insecticidal extracts and dusts, with an active component of approximately 30%. Pyrethrin-I and pyrethrin-II are the most known types of pyrethrins. The pyrethrins further possess four dissimilar active elements, jasmolin I and II and cinerin I and II. The primary use of pyrethrin elements is to kill cockroaches, human lice, beetles, mosquitoes, and other flies. Several “pyrethrin dusts,” used to regulate insects in horticultural farming, are merely 0.3%-0.5% pyrethrins and are utilized at the ratio of up to 50 lb/acre [52]. Usage of other pyrethrin chemical agents is aimed at killing fleas and rice in poultry pens, on cats and dogs, and warehouse weevils and beetles during storage of grains.

Natural pyrethrins are toxins which speedily infiltrate the insect's nerve system whenever it comes into contact [52]. Some minutes after the insecticide has been applied, the insect cannot fly away or move hence the knockdown dose. However, a “knockdown dose” may not necessarily kill as insect enzymes rapidly detoxify the natural pyrethrins. Some pests recover as a result. Carbamates, organophosphates, or synergists such as piperonyl butoxide (PBO) are combined with the pyrethrins as way of delaying the enzyme action, and thus, a lethal dose is ascertained [53].

Despite the suppleness, impermanence, and plasticity of the WHO tube assay for susceptibility, results indicated that knockdown rates in permethrin were different from deltamethrin. Knockdown rates in samples collected during dry season were also significantly different from the ones collected during wet season. Pyrethroids take effect on nerve cell membranes by deferring the closing of the stimulation gate for the sodium ion channel. Type II pyrethroids, as well as deltamethrin, have *α*-cyano groups that prompt a “long-lasting” obstruction of the sodium channel activation gate. This leads to extended penetrability of the neurone to sodium and generates a chain of recurring nerve signals in sensory nerves, sensory organs, and muscles. According to studies conducted, researchers found out that deltamethrin and other type II pyrethroids may also affect ion passages in the nervous system apart from sodium passages, probably because of their phosphorylation nature [30, 54]. Deltamethrin is potent against insects through direct contact and ingestion. Deltamethrin's working principle is considered to be primarily central in action, or at least initiated from the brain. Once poisons last for a few hours in the insects body, the damage that the nervous system suffers is irreversible, this consequently leads to death of insects.

Physiological dysfunction or cellular injury is the immediate effect of amplified free radical concentration in the insect's body. The two sources of reactive oxygen species (ROS) and reactive nitrogen species (RNS) free radicles include endogenous sources (endoplasmic reticulum, phagocytic cells, mitochondria, peroxisomes, etc.) and exogenous sources (heavy metals, pollution, industrial solvents, alcohol, tobacco smoke, transition metals, pesticides, certain drugs like paracetamol, halothane, and radiation). Free radicals can negatively affect a number of vital groups of biotic molecules, for example, proteins, nucleic acids, and lipids, hence changing the standard redox rank resulting to enhanced oxidative stress. Endogenous is the primary source of reactive oxygen species (ROS) as well as organic free radicals, predominantly through bioactivation and metabolism of xenobiotics (such as a carcinogen, drug, or pesticide) or the reactivity of the existing parent compound. Numerous compounds including insecticides and pesticide have been identified to cause free radical production and have the capability to facilitate these kinds of injuries. The destruction to protein, membrane lipids, and DNA is the termination point biomarker of oxidative stress-inducing consequences of insecticides and pesticides. Therefore, the amounts of membrane lipids and proteins which in turn may be determined by climatic patterns and or edaphic factors affect the knockdown rates and patterns by any given insecticidal agents. Among pyrethrins, allethrin is the least poisonous; deltamethrin is the most toxic to aquatic and terrestrial organisms; fenvalerate, permethrin, and cypermethrin are intermediary lethal pyrethroids [15, 52, 55]. Exposure to cessation products is not a problem since deltamethrin is chemically steady. It has a low vapour pressure (2.0 × 10^−6^ Pa or 1.5 × 10^−8^ mmHg at 25°C) and is regarded to be substantially nonvolatile; breathing hazards are therefore expected to be minimal. It is lipophilic (log Po/w 5.43) and insoluble in water but it is readily soluble in organic solvents; hence, it is more portent during rainy seasons when lipid and protein contents in insects may be higher than during dry spells.

Chemical formula for permethrin is 3-phenoxybenzyl (1RS,3RS;1RS,3SR)-3-(2,2-dichlorovinyl)-2,2-dimethyl-). Permethrin bonds firmly to soil and is decomposed mainly by not only microorganisms but also by photolysis. Sixty percent of the permethrin continued to be on an indoor surface alongside a window and open to daylight, after 20 days of exposure [8]. In water column, usual half-life range for permethrin is approximately 19-27 hours. Nevertheless, permethrin can last for more than a year when adsorbed to sediments. Permethrin affects the nervous system of insects making it hypersensitized to excitations from sense organs as nerves denuded to permethrin send series of impulses. This stimulation develops as a result of sodium ions being blocked by permethrin to move from outer to inner parts of the nerve cells. It obstructs sodium channels to disorder the role of neurons and induce muscles to contract, ending in paralysis and demise. Permethrin's mechanism of action is through ingestion or contact and can still function as a slight repellent due to its higher vapour pressure [52].

Bendiocarb, a carbamate caused 100% mortality rates even before the one hour of exposure to WHO insecticidal papers, was over. It works against an extensive variety of irritating insects and vectors of diseases. It kills wasps, mosquitoes, silverfish, fleas, flies, cockroaches, ants, ticks, and the rest of the pests in households, commercial firms, and food stores. In agriculture, it is effective against diverse insects, particularly those that inhabit the soil. Bendiocarb is well used to treat maize seeds and sugar beets and against slugs and snails. Agrochemicals having bendiocarb are prepared as ultra-low volume sprays, dusts, wettable powders, and as granules. Bendiocarb is extremely poisonous if it penetrates the skin or if it is absorbed. Industrial-grade bendiocarb is a noncorrosive, odorless, white crystalline solid matter. Most at risk are persons exposed under environmental conditions of high humidity and temperature since such circumstances enhance speedy penetration of bendiocarb through the skin and thus a higher insect killing and selection pressure agent in hot and humid times. Bendiocarb interrupts the usual operational mode of the nervous system in an insect and may lead to poisoning after ingestion or contact [56]. The chemical neurotransmitter acetylcholine is discharged to transmit nervous system alerts across the nerve synaptic junction. Once the neurotransmitter is released into the junction, it is disassembled by the acetylcholinesterase enzyme, which is essential for nerve to function properly [56]. Acetylcholine builds up whenever there is suppression of the enzyme, leading to hypersensitization of the nervous system [56]. It is through addition of carbamyl moiety to the active spot of the acetylcholinesterase enzyme that bendiocarb is able to interrupt the nervous system. As a result, it hinders acetylcholine from arriving at the active spot, hence inactivation of the enzyme. However, spontaneous hydrolysis is responsible for releasing the carbamyl group, which consequently reverses the interruption and restores nervous coordination activity back to normal [56]. Cholinesterase, a crucial nervous system enzyme, is normally prohibited by bendiocarb, a reaction which can be reversed as well.

Among all insecticides, pyrethroids are the only ones being utilized to treat LLINs, due to their low toxic nature to humans. But all types of insecticidal chemicals such as organochlorides, carbamates, organophosphates, and pyrethroids are often effective during IRS. Due to environmental concerns and a significant number of malaria vectors having developed resistance to organochlorides especially DDT, a majority of East African nations have banned this group of insecticide [31]. Nonvulnerability of mosquitoes to different groups of insecticides can be attributed to several determinants [17, 31, 57]. Firstly, hereditary determinants include the frequency and number of resistance alleles in the insect population, fitness costs, and relative dominance of the characters. Secondly, biotic determinants include life history parameters of the insect, initial population size, and the fitness cost of the homozygous- and heterozygous-resistant phenotypes. Thirdly, reproductive considerations include rate of increase and fluctuations in population size of the mosquitoes. Fourthly, operational considerations include preceding assortment with other insecticides, application techniques of the chemical, insecticide composition, proportion of the population exposed to selective dose, dosage of insecticide absorbed by insects under experiment, and the age of the sampled mosquitoes [31].

Proper legislation and enactment of appropriate laws and regulations are needed for a country to switch from one agrochemical or public health chemicals to another. Nonvulnerability to insecticide by insect vectors is not a strange occurrence. It is a hereditary characteristic that can escalate in the vector community due to the heightened nonvulnerability selection pressure, an attribute that can swiftly proliferate. Permethrin mortality levels were higher during draught while deltamethrin mortality levels were lower during the same period. This means that meteorological drivers such as wind, rainfall, temperatures, atmospheric pressure and humidity, and edaphic factors, namely, drainage, parental rock material, soil type, soil texture, and topography, affect retention capacity of active compounds, insecticidal activities, and eventually intensity of selection pressure forces on mosquitoes [52, 58]. Anatomical pliability and adjustments could have made adult female mosquitoes aged between 3 and 5 days (from reared field larvae) sampled during the dry season nonvulnerable, more so against deltamethrin in which the main cause of damaging biotic free radicals as well as reactive oxygen species (ROS) is autogenous and not allocable to any extraneous or ecologically factor. Diminished rate of permeation of toughened exoskeleton could have caused decelerated knockdown by deltamethrin. Deltamethrin has a higher capacity to vaporize from water due to its Henry's law constant of 1.2 × 10^−4^ atm m^3^/mol at 25°C, compared with other pyrethroids. But permethrin excitation and hypersensitisation of the nervous system by stimuli from sense organs were not to a greater extent, affected by physiological plasticity or exoskeleton thickening. Cyano group in deltamethrin enables its prolonged endurance in the membrane, while permethrin can penetrate easily from the lipid bilayer with its lipophilic quality to easily access cellular subcompartments such as endoplasmic reticulum (ER) membranes which accommodate CYP450s, the iron source useful during DNA oxidizing hydroxyl radicle formation. Therefore, the hydrophobic and hydrophilic tendencies in the voltage-gated sodium channel and plasma membrane as well as in deltamethrin and permethrin chemicals may have had a bearing to levels of selective pressure action, knockdown rates, and resistance levels in female mosquitoes sampled during dry or wet seasons of the year. Chemicals which are stable to climatic and ecological conditions and intraextracellular environs may have reduced selection pressure in insects. Choice of insecticides by insecticide resistance management programmes should also take into consideration the appropriate insecticide in respect to prevailing climatic and ecological conditions because knockdown rate varied in different insecticides and different rainfall seasons.

Different clusters showed different insecticide resistance allele frequencies. These variations may be related to the flow of mutant genes from mosquitoes in the selection pressure foci. Types of soils, terrain, drainage patterns, topography, and climatic factors come into play by reducing or increasing half-lives of the active ingredients in insecticides and agrochemicals hence reduced or increased selection pressure, respectively. Type of species and species resting and feeding behavior determined the intensities of exposure to the insecticidal agents to be genetically selected for. The number of deletion or insertion repeats in a given genome in a mosquito population may also explain the geographical variations in resistance levels. The fact that resistance had been detected earlier in neighbouring Tororo District in Uganda may not necessarily mean that mutant genes originated from there. Only phylogenic studies can fix the question on origin of resistant gene frequencies. Collection of mosquito samples as well as preparation techniques has been found to affect insecticide vulnerability bioassay results. Death rate of female adults collected from the field was between 10 and 15% greater than in *F*_1_ adults nurtured in the insectary from eggs laid by field-collected blood-fed females [59].

## 5. Conclusions and Recommendations

Both phenotypic and genotypic insecticide resistance levels have been confirmed in Teso North and Teso South subcounties in Western Kenya. Insecticide-resistant levels significantly differed in different clusters at different climatic seasons, types of insecticides, and transmission parameters including species composition. Insecticide resistance management practices in Kenya should be fast tracked and harmonized with agricultural sector agrochemical-based activities and legislation and possibly switch to carbamate use in order to ease selection pressure on pyrethroids which are useable in insecticidal nets and indoor residual spray due to their low human toxicity. The implication of such high resistance levels in mosquitoes collected in Teso subcounties is that resistance is likely to persist and or even increase if monomolecules of permethrin and deltamethrin or both continue to be used in all net and nonnet-based mosquito control purposes. Usage of mutually reinforcing piperonyl butoxide (PBO) that prohibits particular enzymes vital in metabolic activities inside mosquito systems and has been integrated into pyrethroid-LLINs to create pyrethroid-PBO nets is an extremely viable option. Entomological surveillance and monitoring should be done regularly in a predictable schedule. Further studies should be done on alternative molecular markers of resistance and sources of selection pressure on malaria vectors.

## Figures and Tables

**Table 1:** Proportions in species composition in each major malaria vector species per cluster and method of collection in Teso North and Teso South subcounties, Western Kenya between 2012 and 2014.

Cluster	*n*	*Anopheles gambiae* sensu stricto	*Anopheles arabiensis*	*Anopheles funestus*
		Proportions %	Proportions %	Proportions %
Kaliwa	491	80.6	19.4	0
Odioi	1041	81.6	18.4	0
Akiriamasit	738	65.7	34.3	0
Kengatunyi	177	79.1	20.9	0
Rwatama	1724	87.2	10.7	2.1

Mean	**4171**	**78.9**	**20.7**	**0.4**

Teso North	2639	77.3	22.0	0.7
Teso South	1532	81.1	18.9	0
Collection method				

Larval collections	2588	77.2	22.8	0
PSC	338	83.3	16.7	0
WET	722	64.1	35.9	0
OPC	36	25	25	50
HLC	605	81.6	18.4	0

**Table 2:** Percentage mortality after 24 h exposure period during WHO tube assay for susceptibility to permethrin, deltamethrin, and bendiocarb in 3-5-day-old mosquitoes from larvae collected from Teso North and Teso South subcounties, Western Kenya, between 2012 and 2014.

Year	2012			2013		2014		Mean^SD^		
Clusters	Different types of insecticides on WHO-treated test papers for tube assay									
	Permethrin	Deltamethrin	Permethrin	Deltamethrin	Bendiocarb	Permethrin	Deltamethrin	Permethrin	Deltamethrin	Bendiocarb
Kaliwa	75^∗^^101^	63^∗^^16^	74^∗^^118^	93^∗^^100^	-	76^∗^^67^	79^∗^^52^	75^±5.4^	78^±15.6^	-
Odioi	37^∗^^99^	56^∗^^34^	79^∗^^103^	89^∗^^100^	100^∗^^61^	56^∗^^101^	53^∗^^100^	57^±19.4^	66^±19.1^	100
Akiriamasit	81^∗^^89^	-	78^∗^^117^	96^∗^^107^	100^∗^^100^	53^∗^^19^	76^∗^^84^	71^±15.4^	75^±11.8^	100
Kengatunyi	87^∗^^107^	66^∗^^44^	91^∗^^102^	99^∗^^116^	-	80^∗^^92^	93^∗^^14^	86^±5.7^	86^±18.3^	-
Rwatama	86^∗^^103^	70^∗^^38^	75^∗^^116^	87^∗^^101^	-	38^∗^^8^	78^∗^^63^	66^±23.1^	78^±8.0^	-

Mean^SD^	73^±19.5^	64^±10.5^	79^±8.2^	93^±6.9^	100	61^±16.7^	76^±14.5^	71	77	100

^∗^Superscripted numbers represented the number of 3-5-day-old female mosquitoes assayed. ^−^Where 3-5-day-old female mosquitoes were not available for exposure to that particular insecticide. WHO criteria for susceptibility are as follows: mortality rates between 98% and 100% indicate full susceptibility; mortality rates between 90% and 97% require further investigation while mortality rates < 90%, the population is considered resistant to the tested insecticides.

**Table 3:** *Kdr* genotypic and allelic frequencies across the clusters, mosquito collection methods, species, and year in mosquito samples collected from Teso North and Teso South subcounties, Busia County, Western Kenya.

Cluster	*n*	SS genotypic frequency	LS genotypic frequency	LL genotypic frequency	S allelic frequency	L allelic frequency
		Proportions %	Proportions %	Proportions %		
Kaliwa	223	91.5	2.7	5.8	0.93	0.07
Odioi	424	76.9	4.7	18.4	0.79	0.21
Akiriamasit	216	76.9	13.9	9.2	0.84	0.16
Kengatunyi	85	57.6	7.1	35.3	0.61	0.39
Rwatama	148	93.9	3.4	2.7	0.95	0.05

Mean	**219.2**	**79.4**	**6.9**	**13.7**	**0.82**	**0.18**

Teso North	449	78.6	9.4	12.0	0.83	0.17
Teso South	647	82.0	4.0	14.0	0.84	0.16
Collection method						

Larval collections	556	80.6	6.1	13.3	0.84	0.16
PSC	9	100	0	0	1.0	0
WET	348	79.0	4.0	17.0	0.81	0.19
OC	—	—	—	—	—	—
HLC	183	83.0	10.4	6.6	0.88	0.12
Species						

*Anopheles gambiae* s.s.	868	81	5.2	13.8	0.84	0.16
*Anopheles arabiensis*	228	80.6	6.2	13.2	0.84	0.16
*Anopheles funestus*	—	—	—	—	—	—
Year						

2012	106	66	5.7	28.3	0.69	0.31
2013	230	83.9	7.0	9.1	0.87	0.13
2014	760	81.6	6.1	12.3	0.85	0.15

**Table 4:** *Kdr* genotypic frequency per subcounty, cluster, major species, and year in female mosquitoes sampled from Teso North and Teso South subcounties in Busia County, Western Kenya, between 2012 and 2014.

Cluster	*n*	Major species	2012			2013			2014		
			SS	LS	LL	SS	LS	LL	SS	LS	LL
Kaliwa	*170*	*An. gambiae*	91	0	9	94	2	4	94	3	3
	*53*	*An. arabiensis*	0	0	100	93	0	7	82	5	13
Odioi	*354*	*An. gambiae*	61	4	35	82	5	13	79	3	18
	*70*	*An. arabiensis*	83	10	17	40	20	40	73	11	16
Akiriamasit	*151*	*An. gambiae*	42	8	50	88	9	3	79	12	9
	*65*	*An. arabiensis*	80	0	20	67	29	4	77	18	5
Kengatunyi	*57*	*An. gambiae*	0	20	80	57	7	36	50	5	45
	*28*	*An. arabiensis*	0	0	0	0	0	0	79	7	14
Rwatama	*136*	*An. gambiae*	87	13	0	88	4	8	96	2	2
	*12*	*An. arabiensis*	100	0	0	100	0	0	100	0	0
Teso North	*344*	*An. gambiae*	56	13	31	82	7	11	81	7	12
	*105*	*An. arabiensis*	90	0	10	68	27	5	80	12	8
Teso South	*524*	*An. gambiae*	67	3	30	88	4	8	84	3	13
	*123*	*An. arabiensis*	71	0	29	88	3	9	78	8	14

**Table 5:** Knockdown times for 50 and 95% of the susceptible Kisumu *Anopheles gambiae* strain and the Teso North and Teso South *F*_0_*Anopheles gambiae* to permethrin, deltamethrin, and bendiocarb diagnostic concentrations.

			Susceptible Kisumu Asembo *Anopheles gambiae* strain				Teso North and South *F*_0_*Anopheles gambiae* samples			
Diagnostic concentrations	Clusters	*n*	KDT_50_ [CI] (min)	KDT_95_ [CI] (min)	Mortality after 60 min (%)	*n*	KDT_50_ [CI] (min)	KDT_95_ [CI] (min)	Mortality after 24 h (%)	KDT_50_ R
0.75% permethrin	Kaliwa	100	**14.155** [6.207-20.372]	**32.462** [25.469-50.817]	100	95	**41.285** [38.579-44.369]	**103.363** [88.043-129.284]	75	2.9
	Odioi	100	**17.658** [4.133-27.790]	**58.565** [34.896-1259.225]	100	100	**46.607** [36.379-71.682]	**189.492** [104.482-1062.44]	57	2.6
	Akiriamasit	100	**13.977** [3.707-21.112]	**61.544** [37.499-466.702]	100	75	**24.689** [19.390-30.196]	**66.066** [48.540-128.794]	71	1.8
	Kengatunyi	100	**8.189** [5.947-10.563]	**27.813** [21.837-39.676]	100	100	**36.397** [34.402-38.442]	**74.654** [67.294-85.557]	86	4.4
	Rwatama	100	**14.982** [12.625-17.192]	**35.886** [29.288-48.448]	100	76	**37.085** [34.536-39.788]	**84.719** [73.667-102.885]	66	2.5
0.05% deltamethrin	Kaliwa	100	**17.590** [15.962-19.036]	**26.432** [23.914-30.975]	100	76	**30.930** [23.644-41.566]	**86.182** [56.925-292.337]	78	1.8
	Odioi	100	**5.780** [3.534-7.794]	**31.050** [25.978-39.834]	100	78	**31.757** [29.521-34.007]	**71.317** [63.207-83.777]	66	5.5
	Akiriamasit	100	**13.256** [4.132-19.714]	**62.378** [38.849-328.090]	100	96	**35.448** [30.809-40.298]	**70.829** [58.069-102.798]	75	2.7
	Kengatunyi	100	**2.698** [0.722-4.916]	**24.154** [18.974-32.751]	100	71	**22.204** [20.821-25.139]	**49.690** [46.172-52.962]	86	8.2
	Rwatama	100	**17.012** [15.200-19.304]	**50.773** [31.357-402.770]	100	67	**30.423** [27.859-33.198]	**80.692** [70.893-97.137]	78	1.8
0.1% bendiocarb	Odioi	100	**5.432** [0.679-10.006]	**51.612** [33.976-172.401]	100	61	**20.543** [18.570-23.086]	**58.895** [53.951-67.980]	100	3.8
	Akiriamasit	100	**5.439** [0.258-10.444]	**39.386** [25.578-168.087]	100	100	**24.886** [17.223-31.783]	**48.181** [36.636-100.030]	100	4.6

*n*: sample size; CI: confidence interval at 50 and 95%; KDT_50_: knockdown times for 50% of exposed mosquitoes; KDT_95_: knockdown times for 95% of exposed mosquitoes; KDT_50_ R: ratio KDT_50_ Teso North and Teso South *F*_0_ female mosquito samples/KDT_50_ susceptible Kisumu Asembo *Anopheles gambiae* strain; min: time in minutes.

## Data Availability

The data used to support the findings of this study are available from the corresponding author upon request.

## Abbreviations

ACTs: Artemisinin-based combination therapies
AR: *Anopheles arabiensis*
DDT: Dichlorodiphenyltrichloroethane
DNA: Deoxyribonucleic acid
ELISA: Enzyme-linked immunosorbent assay
GA: *Anopheles gambiae*
HLC: Human landing catch
IPTp: Intermittent preventive treatment in pregnancy
IRM: Insecticide resistance management
IRS: Indoor residual spraying
ITN: Insecticide-treated net
JH: Juvenile hormone
*Kdr*: Knockdown resistance gene
LL: Homozygous recessive or susceptible
LLIN: Long-lasting insecticidal net
LS: Heterozygous resistant
OPC: Outdoor pot collection
PBO: Piperonyl butoxide
PCR: Polymerase chain reaction
PMI: President's malaria initiative
PSC: Pyrethrum spray catch
ROS: Reactive oxygen species
SS: Homozygous resistant
UN: Universal primer
WET: Window exit trap
WHO: World Health Organization.
